# Lung Adenocarcinoma Mimicking a Bilateral Cavitary Pneumonia

**DOI:** 10.5334/jbsr.2825

**Published:** 2022-06-08

**Authors:** Cédric Mahiat, Benoît Colinet, Pierre-Antoine Poncelet

**Affiliations:** 1 Grand Hôpital de Charleroi, BE

**Keywords:** lung cavities, lung adenocarcinoma, pneumonia, thoracic imaging, computed tomography

## Abstract

**Teaching Point:** Failure to recognize unusual radiological presentations of some lung adenocarcinomas can lead to misdiagnosis and/or delay appropriate treatment.

## Case History

**Figure 1:**
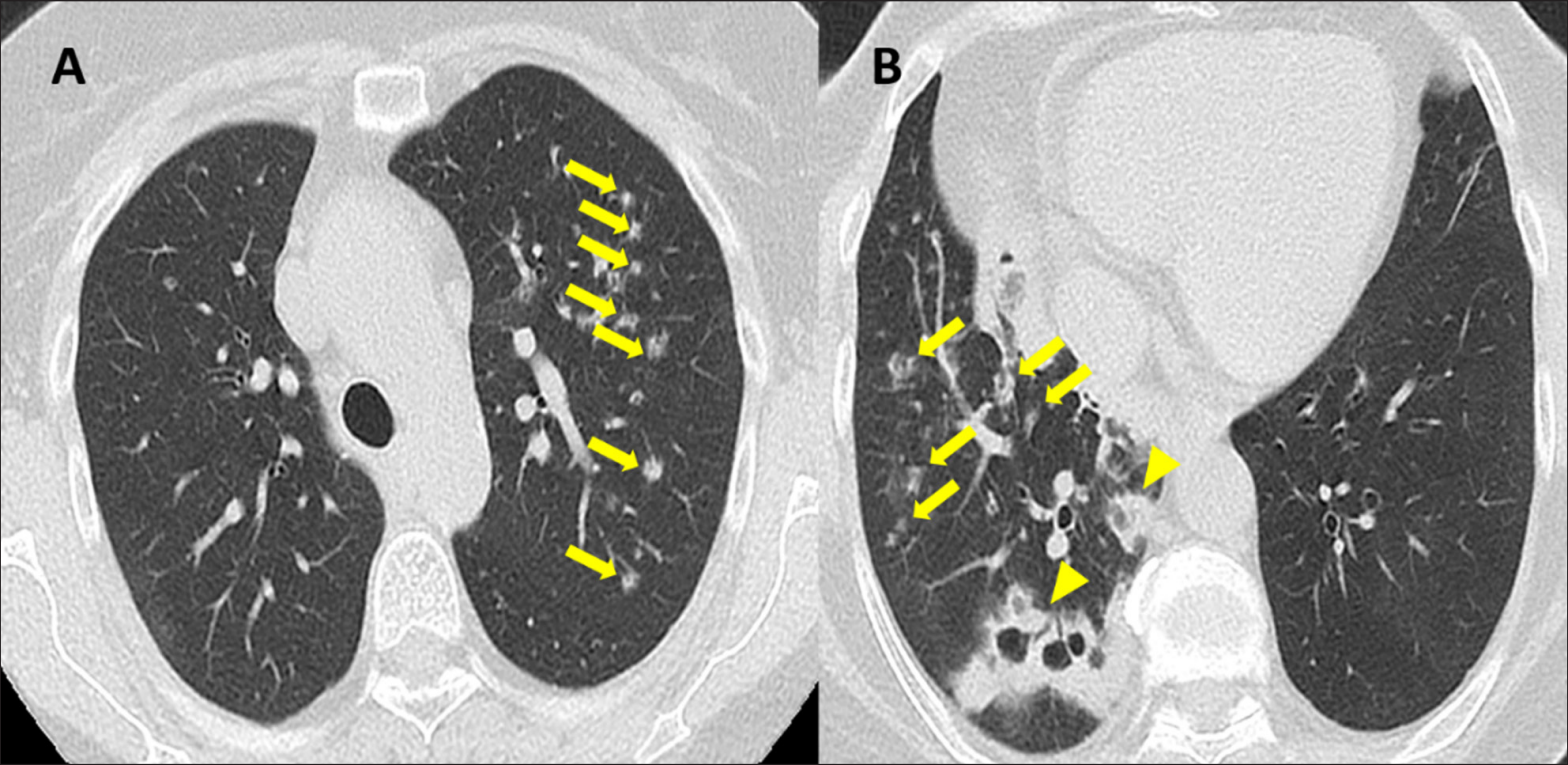


A 60-year-old female without smoking history presented with persisting dry cough. A chest computed tomography (CT) scan performed at the first visit showed multiple bilateral nodules with cavitation and surrounding ground-glass opacities especially in the left upper lobe and the right lower lobe (arrows, Figure 1A–B), and areas of consolidation with necrotizing cavities in the right lower lobe (arrowheads, Figure 1B).

A bronchoscopy was performed for microbiologic evaluation. Nucleic acid amplification test (NAAT) was positive for *Mycobacterium tuberculosis* but not confirmed in culture due to insufficient sample size. A mycobacterial dedicated four-drug regimen was prescribed.

**Figure 2:**
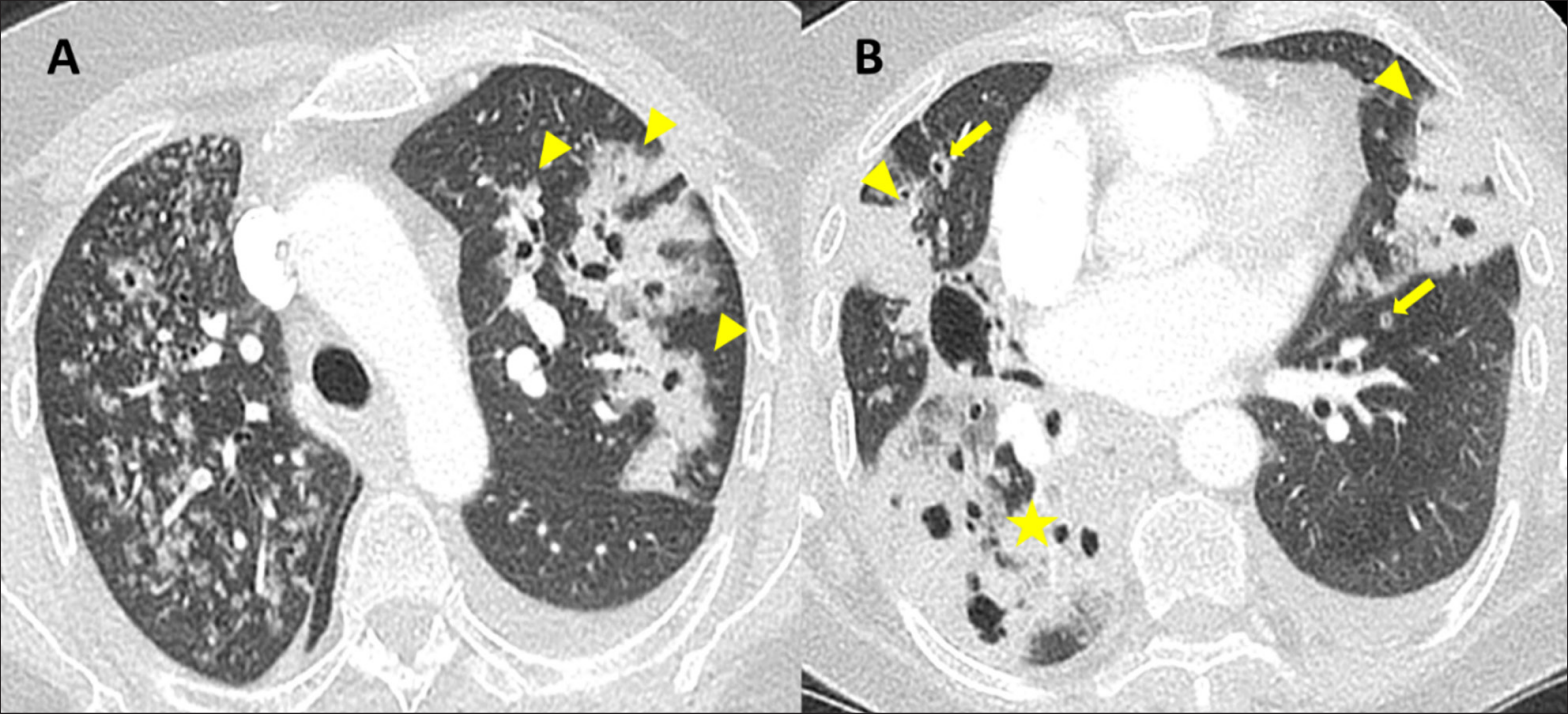


Four months later, the patient presented with an unprovoked bilateral pulmonary embolism. On the chest CT scan, the bilateral cavitary pneumonia had worsened with enlargement of cavitary nodules to confluent consolidation areas with necrotizing cavities in the left upper lobe (arrowheads, Figure 2A) and the appearance of multiples centrilobular nodules in the right upper lobe suggesting bronchogenic spread (Figure 2A). There was also enlargement of the necrotizing right lower lobe consolidations (star, Figure 2B), and the occurrence of cavitating nodules (arrows, Figure 2B) and necrotizing lung consolidations (arrowheads, Figure 2B) in the middle lobe, left lower lobe, and the lingula.

The anti-tuberculosis regimen was withheld. New bacteriological samples were obtained. NAAT was negative for *Mycobacterium tuberculosis*, as well as the mycobacterial culture. Biological autoimmune disease screening was negative. Given the progression, surgical lung biopsies were performed on the right lung. They showed a malignant epithelial proliferation characterized by papillary glandular structures, consistent with the diagnosis of lung adenocarcinoma. There was no mediastinal lymph node invasion nor distant metastasis. The patient then underwent standard of care systemic treatment combining chemotherapy and immunotherapy in first line setting.

## Comment

Cavitary lung lesions can be observed in infectious, inflammatory, or neoplastic diseases [1]. Radiological features, like the wall thickness, the presence or absence of perilesional condensations, and centrilobular nodules may suggest the benign or malignant nature of the lesions [1]. However, as these features are not entirely specific, the clinical context, such as the presence of fever, weight loss, and acute versus chronic onset, and paraclinical findings must always be taken into consideration [1]. Our patient presented with multiple lung cavities surrounded by areas of ground glass opacities or consolidations, which could suggest an infectious disease. This was corroborated by a false positive *Mycobacterium tuberculosis* NAAT. Nevertheless, the lack of fever and the progression of the lesions despite the antimicrobial treatment should direct to an alternative diagnosis, such as neoplastic disease.

In case of radio-clinical discordance or poor evolution despite the supposedly appropriate treatment, the initial diagnosis must be questioned and further investigations, including surgical lung biopsies if relevant, should be considered.
